# Edible bird's nest alleviates pneumonia caused by tobacco smoke inhalation through the TNFR1/NF‐κB/NLRP3 pathway

**DOI:** 10.1002/fsn3.4080

**Published:** 2024-04-01

**Authors:** Ran Bi, Dan Zhang, Rui Quan, Xiaoxian Lin, Wen Zhang, Chuangang Li, Man Yuan, Bing Fang, Dongliang Wang, Yixuan Li

**Affiliations:** ^1^ Key Laboratory of Precision Nutrition and Food Quality, Department of Nutrition and Health China Agricultural University Beijing China; ^2^ Hebei Edible Bird's Nest Fresh Stew Technology Innovation Center Langfang China; ^3^ Key Laboratory of Function Dairy, Co‐Constructed by Ministry of Education and Beijing Municipality, College of Food Science and Nutritional Engineering China Agricultural University Beijing China

**Keywords:** dietary therapy, edible bird's nest, NF‐κB signaling, pulmonary inflammation, tobacco hazard

## Abstract

Exposure to cigarette smoke directly damages the lungs and causes lung inflammation. The anti‐inflammatory properties of edible bird's nest (EBN) have been reported. We aimed to determine the effect of EBN on pneumonia in a mouse model exposed to cigarette smoke. Fifty BALB/c mice were randomly divided into control, model, positive drug, low‐dose EBN, and high‐dose EBN groups (*n* = 10 each). Except for the control group, the mice in each group were exposed to four cigarettes once a day for 8 days. In addition, we validated the effects of EBN on A549 cells and investigated the mechanism by which EBN alleviates lung inflammation. Edible bird's nest (EBN) could alleviate the structural damage of lung tissue and the smoke‐induced inflammatory response in mice. The best effect was observed at the high dose of EBN (0.019 g). The mice treated with EBN had a stronger ability than those in the model group to resist cigarette smoke stimulation, as indicated by a decrease in serum and lung inflammatory markers (interleukin 6 [IL‐6], tumor necrosis factor‐α [TNF‐α], and interleukin 8 [IL‐8]), an increase in serum interleukin 10 (IL‐10) levels, and a decrease in the expression of inflammasome NOD‐like receptor pyrin 3 (NLRP3). In addition, our cell experiments showed that EBN attenuated cigarette smoke‐induced pulmonary inflammation mainly by inhibiting the tumor necrosis factor receptor 1 (TNFR1)/nuclear factor‐kappa B (NF‐κB)/NLRP3 pathway. These findings provide theoretical evidence for the positive nutritional qualities of EBN for the lung by demonstrating that it inhibits the TNFR1/NF‐κB/NLRP3 signaling pathway, which prevents the development of cigarette smoke‐induced pulmonary inflammation.

## Graphical abstract

Edible bird's nest (EBN) promotes epithelial cell proliferation and viability, which reduces alveolar epithelial damage and lower levels of inflammation in the lungs and body. Edible bird's nest can effectively inhibit the expression of NOD‐like receptor pyrin 3 (NLRP3) inflammasome, thereby effectively controlling the smoke‐induced pulmonary inflammatory response. Edible bird's nest reduces the activation of the NLRP3 inflammasome by inhibiting the activation of the nuclear factor‐kappa B (NF‐κB) pathway during inflammatory injury.

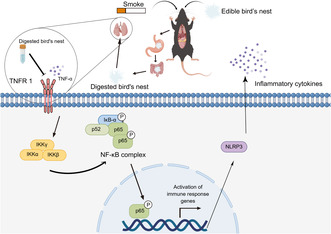

## 1 INTRODUCTION

Tobacco use is considered to be the single biggest cause of preventable disease and premature death worldwide. The organs that are most directly damaged by smoking are the lungs. Tobacco smoke causes chronic obstructive pulmonary disease (COPD), respiratory infection, tuberculosis, and a variety of interstitial lung diseases and other respiratory diseases (Strzelak et al., 2018). Most cigarette compounds adversely affect respiratory tract cells (Smith & Hansch, 2000). Nonsmokers exposed to secondhand smoke also have an increased risk of asthma, lung cancer, and coronary heart disease, among others (Eisner, 2010). Secondhand smoke can increase the risk of lung cancer by 20%–30%. According to the World Health Organization (WHO), 1.2 million nonsmokers die yearly from diseases caused by secondhand smoke exposure (Semple et al., 2022).

Inflammation is a process that limits the development of damage and allows the body to repair itself. However, excessive inflammation can also cause different degrees of damage to the body (Oishi & Manabe, 2018). The pro‐inflammatory properties of cigarette smoke have been well documented (Hellermann et al., 2002; Mio et al., 1997). Smoking modulates the inflammatory response elicited by respiratory epithelial cells by regulating the production of a plethora of potent pro‐inflammatory cytokines and chemokines while continuously recruiting macrophages and neutrophils, thereby further compromising lung structure and function and respiratory immune system integrity (Rom et al., 2013). Additionally, smoking results in the release and inhibition of pro‐inflammatory and anti‐inflammatory mediators, and smoking has been demonstrated to enhance the production of pro‐inflammatory cytokines, including tumor necrosis factor‐α (TNF‐α), interleukin (IL)‐1, IL‐6, and IL‐8, while reducing the levels of anti‐inflammatory cytokines such as interleukin 10 (IL‐10) (Arnson et al., 2010). Furthermore, the increase in circulating Immunoglobulin E (IgE) and cytokines in smokers triggers a chronic systemic inflammatory response, which is associated with a large network of pulmonary and systemic cytokines (Arnson et al., 2010). The systemic inflammatory response triggered by cigarette smoke exposure is characterized by stimulation of the hematopoietic system, especially the bone marrow, leading to the release of leukocytes and platelets into the circulation, which is attributed to the relative increase in circulating polymorphonuclear neutrophil counts in smokers (He et al., 2016). One of the key mechanisms behind the smoking‐induced activation of inflammatory cells is the nuclear factor‐kappa B (NF‐κB) pathway (Gonçalves et al., 2011), which is activated by cigarette smoke and extracts of cigarette smoke in a variety of cells of the immune system (Rom et al., 2013). The NOD‐like receptor pyrin 3 (NLRP3) inflammasome is a key regulator of the host innate immune response, which can recognize a variety of pathogenic microorganisms and stress‐related endogenous signaling molecules and play a role in amplifying the immune response. Toll‐like receptor 4 (TLR4) or tumor necrosis factor receptor (TNFR) activation induces NF‐κB signaling, leading to elevated NLRP3 expression, which in turn is activated indirectly by numerous signals. NLRP3 can further activate caspase‐1 to mature interleukin 1β (IL‐1β) and interleukin 18 (IL‐18) (He et al., 2016).

Edible bird's nest (EBN) has garnered significant attention due to standing as a nutrient‐dense food with a wide array of physiological and pharmacological effects. Since the Tang Dynasty (618–907 AD), EBN has been considered to be able to moisten the lungs. It has been used to treat many acute and chronic respiratory diseases (Haghani et al., 2016). Zeng et al. (2022) found that EBN could regulate the secretion of cytokines in mice with lung Yin deficiency to inhibit inflammation, slow down the immune stress response of splenic lymphocytes, and inhibit the apoptosis of splenic lymphocytes, thus regulating the immune balance and alleviating lung injury in mice. Haghani et al. (2016) reported that EBN may affect the viral NA and NS1 genes to inhibit influenza virus while regulating the immune response through cell‐mediated immune activation, thus effectively improving the ability to resist influenza infection, confirming the effectiveness of EBN in the treatment of respiratory diseases. Numerous investigations have revealed that EBN is a multicomponent complex meal, and that its anti‐inflammatory qualities may be attributed to the synergistic effects of its different bioactive ingredients (Jacobs et al., 2009). In vivo, EBN was shown to reduce inflammatory factors, such as IL‐1β, IL‐6, and TNF‐α, in a C57BL/6 mouse model while altering the messenger RNA (mRNA) expression of cyclooxygenase 2 (COX‐2) and strongly reducing NF‐κB signaling (Lai et al., 2022). Moreover, EBN exerts anti‐inflammatory activity by regulating the antioxidant response in the liver and reducing the expression of inflammatory markers IL‐6 and TNF‐α and the inflammatory gene *Nf‐κb1* in a high‐fat diet‐induced rat model of oxidative stress and inflammation (Yida et al., 2015). In vitro, EBN alleviates ultraviolet (UV)‐ and arid environment‐induced cellular oxidative stress, cell death, and DNA damage in human HaCaT keratinocytes and three‐dimensional (3D) epithelium equivalents (Wang et al., 2023). Recent studies have revealed that EBN also has anti‐inflammatory effects, promotes fetal brain development, improves human memory, exerts antioxidant effects, and regulates intestinal flora (Zeng et al., 2022). Vimala et al. (2012) found that EBN can affect the proliferation of human colon cancer cells and the release of TNF‐α in RAW264.7 macrophages, indicating that EBN has anti‐inflammatory effects. Guo et al. (2006) showed that EBN aqueous extract could inhibit the hemagglutination reaction of red blood cells (RBCs), neutralize virus infectivity, and inhibit influenza virus infection of canine kidney (CDK) cells. Wong, Chan, Wu, Lam, et al. (2018); Wong, Chan, Wu, Poon, et al. (2018) confirmed the existence of acidic mammalian chitinase (AMCase), which plays a beneficial role in inflammatory lung disease, in EBN (Mazur et al., 2021). Haghani et al. (2016) pointed out that sialic acid in EBN can act as a regulator of the immune system, affecting the flow resistance of mucus and thereby eliminating bacteria, viruses, and other harmful microorganisms.

While there is no evidence to suggest that EBN can alleviate the adverse effects of tobacco use in cultured keratinocytes and mice with dermatitis, EBN treatment resulted in the inhibition of NF‐κB pathway protein expression (Lai et al., 2022). Consequently, we hypothesized that EBN, in conjunction with its anti‐inflammatory qualities, would lessen the pulmonary inflammation that smoke causes in mice. To further explore the anti‐inflammatory activity of EBN, we initially evaluated the protective effect of EBN against cigarette smoke‐induced pneumonia in mice, followed by verification of its anti‐inflammatory properties using human alveolar basal epithelial A549 cells as a model for pneumonia. Finally, we clarified the anti‐inflammatory mechanism of EBN, providing a theoretical basis for the prevention of cigarette‐induced lung injury by EBN products, a scientific basis for the further development of special medical food based on EBN, and a molecular target for the development of precise drugs for the intervention of chronic lung diseases in the future.

## 2 MATERIALS AND METHODS

### 2.1 EBN sample preparation and simulated digestion

Dried EBN imported from Indonesia was first cleaned with tap water. After drying, 5 g of EBN was dissolved in 95 mL of pure water, and the final EBN was obtained by boiling at 95°C for 15 min. Digestion was performed as previously described in the literature, with slight modifications (Gil‐Izquierdo et al., 2002). The simulated gastric fluid (SGF) contained 0.0122 g/L KH_2_PO_4_, 0.0514 g/L KCl, 0.0009 g/L MgCl_2_, 0.2100 g/L NaHCO_3_, and 0.0039 g/L (NH_4_)_2_CO_3_. It was mixed with a juicer, after which pepsin was added to achieve a final enzymatic activity of 4000 U/mg. The pH of the mixture was adjusted to 2.0 using 1.0 M HCl, and the mixture was placed in a 37°C incubator for 2 h with shaking at 300 rpm. The simulated small intestine fluid (SIF) contained 0.0108 g/L KH_2_PO_4_, 0.0506 g/L KCl, 0.0031 g/L MgCl_2_, 0.7140 g/L NaHCO_3_, 0.2244 g/L NaCl, and 0.0067 g/L CaCl_2_. After gastric digestion, the mixture was mixed 1:1 with SIF, and then trypsin was added to achieve a final enzymatic activity of 100 U/mL. The pH of the mixture was adjusted to 7.0 using 1.0 M NaOH, and the mixture was placed in a 37°C incubator for 2 h with shaking at 300 rpm. Finally, it was boiled in a water bath for 10 min to inactivate the enzyme. After complete freezing, it was freeze‐dried for 48 h to produce the simulated digested EBN dry powder, which was stored at −20°C.

### 2.2 Animal experiments

BALB/c mice aged 7 weeks were obtained from Beijing Vital River Laboratory Animal Technology Co., Ltd. (Beijing, China) and reared at Pony Testing International Group Co., Ltd. (Beijing, China). The experimental animals were kept in animal facilities in standard laboratory conditions with free access to food and water for a week to acclimate before the experiment. The experiment was conducted following the ethical review regulations for animal experiments of Pony Testing International Group Co., Ltd. (Approval No. PONY‐2022‐FL‐13). The mouse model of cigarette‐induced pneumonia was generated, as previously described with modifications (Zeng et al., 2022). Fifty BALB/c mice were randomly divided into the following five groups (*n* = 10 mice per group): (i) the control group, (ii) the model group, (iii) the positive drug group (0.4 mL/10 g.bw), (iv) the low‐dose EBN group (0.008 g EBN), and (v) the high‐dose EBN group (0.019 g). Cigarettes were purchased from Shanghai Tobacco Group Co., Ltd. (Shanghai, China). Except for the control group, the mice were smoked in a glass smoker (10 mice smoked by four sticks) once a day for 8 consecutive days, 10–12 min each time. From the beginning of modeling, each group of mice was given a gavage simultaneously for 15 consecutive days. The control and model groups were treated with normal saline by gavage, and the positive drug group was treated with Baihe gujin decoction (JST68000061; Tongrentang, Beijing, China). The EBN dry powder was dissolved into saline and administered by gavage. Body weights of mice were recorded on the first, seventh, and last days. Animals were euthanized, and blood and tissues were collected for the examination of protein expression and histology.

### 2.3 Histopathology

The left lung lobe was fixed with 4% paraformaldehyde (PFA) (BL539A; Biosharp, Beijing, China) solution; cleared in xylene (S1763; Keao, Beijing, China) for 25 min; soaked in 50%, 80%, 90%, 95%, and 100% ethanol (213‐3; LookChem, Tianjin, China) for 5 min each time; rinsed with running water for 10 min; embedded in paraffin (HistoCore Arcadia C; Leica, Shanghai, China); and sectioned with a tissue slicer (Leica CM30505; Leica, Nussloch, Germany). Tissue sections were dewaxed, stained with hematoxylin for 15 min, rinsed with running water for 2 h, stained with eosin solution for 2 min, and sealed. The tissue slices were observed using an optical microscope (XSP‐8CA; Yoke Instrument, Shanghai, China). The Smith lung injury scores (Eltzschig et al., 2014) were determined to evaluate lung injury in a blinded manner.

### 2.4 Immunohistochemical staining

In our immunohistochemical (IHC) staining experiment, we adopted the methodology outlined by He et al. (2022). Lung sections were sealed at room temperature for 30 min with 10% goat serum, incubated overnight at 4°C with a primary anti‐NLRP3 antibody (1:200 dilution, DF7438; Affinity, Jiangsu, China), and incubated at room temperature for 45 min with horseradish peroxidase (HRP)‐conjugated goat anti‐rabbit IgG(H + L) (1:2000 dilution, A0208; Beyotime). Then the sections were rinsed and treated with diaminobenzidine (DAB) for 15 min. NLRP3 staining was analyzed under an optical microscope (XSP‐8CA; Yoke Instrument, Shanghai, China). ImageJ software (version V1.8.0‐172, 64‐bit) was used to quantify the optical density.

### 2.5 Western blot

For Western blot, we referred to the method described by Kim et al. (2019). Proteins were extracted from cultured cells with radioimmunoprecipitation assay (RIPA) lysis buffer (89901; Thermo Scientific) containing 1% phenylmethylsulfonyl fluoride (PMSF) (P0100; Solarbio) and 1% protein phosphatase inhibitor (P1260; Solarbio). Next, 5× sodium dodecyl sulfate‐polyacrylamide gel electropheresis (SDS‐PAGE) loading buffer containing dithiothreitol (DTT) (P1040; Solarbio) was added, and the samples were boiled for 10 min. The proteins were separated using SDS‐PAGE at 80 V for 30 min and 120 V for 90 min. The proteins were transferred to polyvinylidene fluoride (PVDF) membranes (Millipore, China) using a wet transfer method at a constant current of 200 mA for 120 min. The membranes were incubated in 5% bovine serum albumin (BSA) at room temperature for 1 h, with anti‐TNFR1 (ab259817; Abcam), anti‐phospho‐IκBα (9246S; Cell Signaling Technology), anti‐NF‐κB‐phospho‐p65 (3033S; Cell Signaling Technology), anti‐IκBα (4812S; Cell Signaling Technology), anti‐NF‐κB p65 (ab16502; Abcam), anti‐β‐actin (3700S; Cell Signaling Technology), and anti‐NLRP3 (DF7438; Affinity) overnight at 4°C, and with horseradish peroxidase (HRP)‐conjugated goat anti‐rabbit Immunoglobulin G (IgG) (H + L) (1:5000 dilution, A0208; Beyotime) at room temperature for 1 h. Primary antibodies were diluted 1:1000. Finally, the protein bands were visualized using a luminescent image analyzer (Amersham Imager 600; GE Healthcare Bio‐Sciences AB, Uppsala, Sweden).

### 2.6 Immunofluorescence staining

For immunofluorescence staining, we referred to the method described by Kim et al. (2019). Suspended cells were inoculated in confocal culture dishes and incubated for 24 h. The cells were treated with EBN for 24 h and then with TNF‐α (300‐01A‐50UG; PeproTech) for 2 h. Then the cells were fixed with 4% paraformaldehyde (FB24243; Feimobio), permeabilized with 0.5% Triton X‐100, blocked with 5% BSA for 2 h, incubated overnight with the primary antibody in a wet box at 4°C, incubated for 1 h at 37°C with the secondary antibody (A0468; Beyotime) in the dark, and incubated with DAPI (4′,6‐diamidino‐2‐phenylindole) (C0065; Solarbio) for 10 min in the dark. The dishes were sealed with a sealing solution containing an anti‐fluorescence quenching sealing solution (P0126; Beyotime), and the images were observed under a confocal laser scanning microscope (LSM900; Zeiss, Nussloch, Germany).

### 2.7 Enzyme‐linked immunosorbent assay

We performed the enzyme‐linked immunosorbent assay (ELISA) strictly in compliance with the kit's instructions. The lung tissues (0.1 mg) were homogenized in 1 mL phosphate‐buffered saline (PBS) using a tissue homogenizer (FastPrep‐24; MP Biomedicals, Solon, OH, USA). After centrifugation, the supernatant was collected and stored at −80°C until further analysis. Mouse blood was collected in anticoagulant tubes, centrifuged (1000 *g*, 30 min), and stored at −80°C. Cell culture supernatant was centrifuged (300 *g*, 10 min) and stored at −80°C. Repeated freezing–thawing of samples was avoided. Frozen samples were thawed slowly at room temperature and mixed gently. Lung tissue was assayed for IL‐8 (KA10943; Keao) and TNF‐α (EK282HS/3‐96; MultiSciences), and serum was assayed for IL‐6 (EK206HS‐96; MultiSciences), TNF‐α (EK282HS/3‐96; MultiSciences), and IL‐10 (EK210/4‐96; MultiSciences). Furthermore, cell culture supernatant was assayed for IL‐6 (EK106HS‐96; MultiSciences) and IL‐8 (HY‐H0008; Sinoukbio), and cell protein extract was assayed for IL‐10 (EK110HS‐96; MultiSciences).

### 2.8 Cell culture and cell proliferation activity test

In terms of cell culture, we used the method that was used by Tang et al. (2014). The human A549 cell line was purchased from Procell Life Science & Technology Co., Ltd. (Wuhan, China). The cells were cultured in special culture medium (CM‐0016; Procell) at 37°C in an incubator with 5% carbon dioxide (CO_2_). When the cells reached 90% confluence, they were treated with 0.25% trypsin and split. Trypsinized A549 cells were inoculated into a 96‐well plate at a density of 8.0 × 10^3^ cells/well or into a six‐well plate at a density of 1.0 × 10^6^ cells/well and left to attach for 24 h. Then, cells in the experimental group were treated with different concentrations of simulated digested EBN (0–1000 mg/mL) for 24 h and then with TNF‐α (10 ng/mL) for 2 h. The control group was treated with the same amount of nutrient medium.

We meticulously followed the guidelines provided in the instruction manual of the Cell Counting Kit‐8 (CCK8) (CK04; Dojindo) for the cell proliferation activity test. A549 cells in 96‐well plates were treated with different concentrations (0–1000 ng/mL) of TNF‐α for 2 h or 24 h or with different concentrations (0–1000 mg/mL) of simulated digested EBN for 24 h. Alternatively, the cells were treated with simulated digested EBN at different concentrations (0–1000 mg/mL) for 24 h, followed by TNF‐α (10 ng/mL) for 2 h. The control group was treated with the same amount of nutrient medium. After adding 10 μL of CCK8 solution to each well, the plates were incubated in an incubator at 37°C for 1–4 h. Finally, the absorbance at 450 nm was measured with a multifunction microporous detector (16113019; BioTek Instruments, Winooski, Vermont, USA).

### 2.9 Statistical analysis

In our data analysis approach, we relied on the literature as a reference standard to guide our methodologies and techniques (Dinh et al., 2020). All results are expressed as mean ± standard deviation (SD). Three or more groups were compared using one‐way analysis of variance (ANOVA) with Tukey's post hoc correction. A value of *p* ≤ .05 was considered statistically significant (*p ≤ .05, **p ≤ .01, ***p ≤ .001, and ****p ≤ .0001). All figures showing results were plotted using GraphPad Prism 9 (version 9.0.0(121)), and the figure showing functional mechanisms was plotted using Figdraw (Export ID: SUIAP733e7, https://www.figdraw.com/static/index.html#/paint_index).

## 3 RESULTS

### 3.1 EBN reduces lung damage caused by cigarette smoke

**FIGURE 1:**
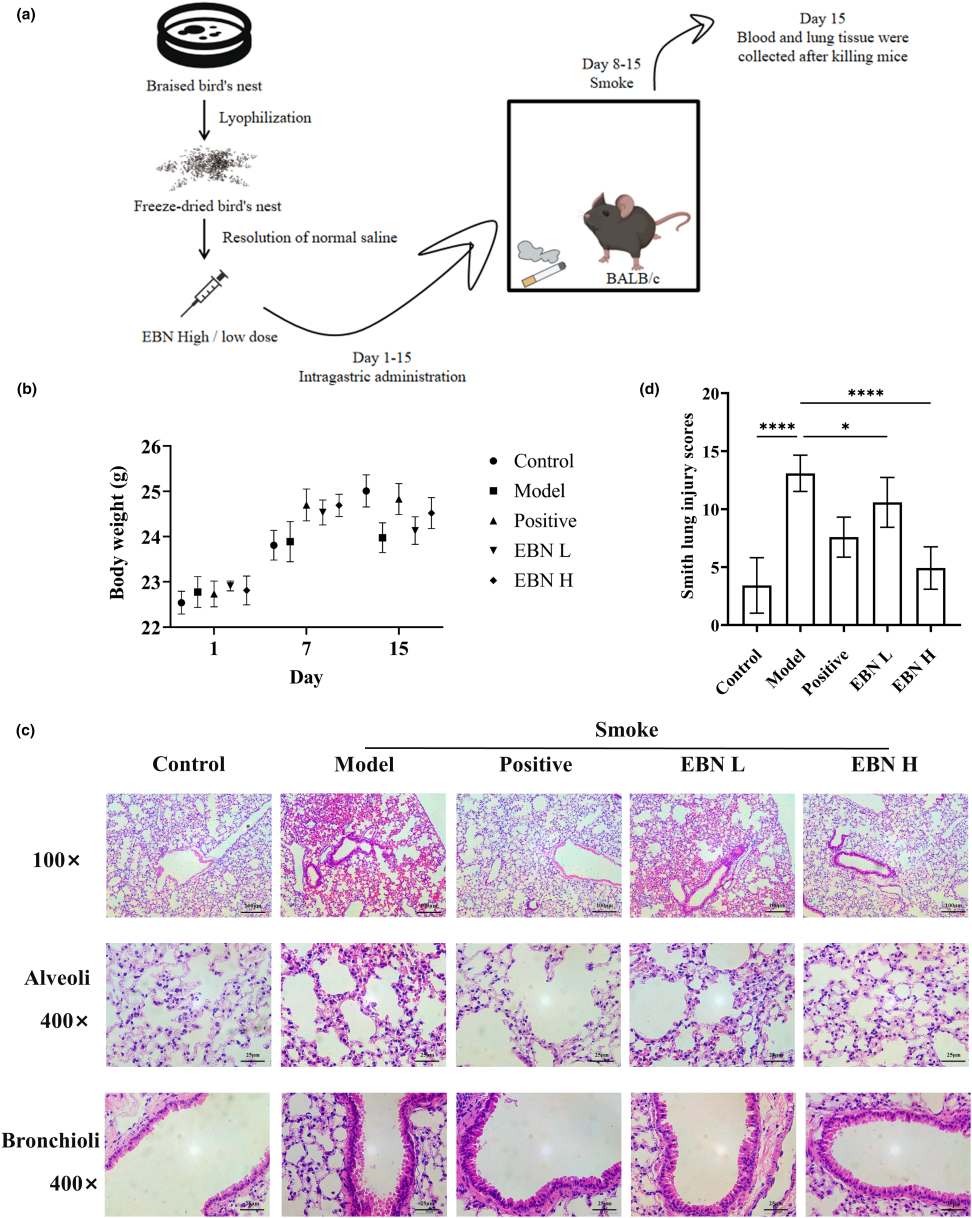
Ingestion of EBN can alleviate lung damage caused by cigarette smoke. (a) Operation flowchart and research design; *n* = 10 animals per group. (b) Body weight of mice; *n* = 10. (c) H&E staining of lung tissue. Magnification, 400×. (d) Smith lung injury score based on H&E staining. Data are expressed as mean ± SD; *n* = 12. Statistical analysis was conducted using nonparametric one‐way ANOVA. Top scale bar = 100 μm; middle and bottom scale bars = 25 μm. Con, the control group; Model, the model group; Positive, the positive drug group (0.4 mL/10 g.bw); EBN L, the low‐dose EBN group (0.008 g EBN); EBN H, the high‐dose EBN group (0.019 g). *p ≤ .05, ****p ≤ .0001.

To test the effect of EBN on inflammation caused by cigarette smoke, we created a model of cigarette smoke‐induced lung inflammation in BALB/c mice (Figure 1a). After inhalation of cigarette smoke, mice developed a cough, weight loss, and showed reduced activity, which was significantly ameliorated by oral EBN (Figure 1b).

Our results (Figure 1c) show that in smoking‐induced chronic bronchitis, damage occurs in the large airways, further leading to the narrowing of the airways with cough, sputum production, and dyspnea. Airway walls are infiltrated by inflammatory cells, followed by scarring, remodeling, wall thickening, and even more airway narrowing. The results of the terminal bronchioles showed that cigarette use disrupted the neat arrangement of cilia in the trachea and that there was a high degree of inflammatory cell infiltration. EBN alleviates airway inflammation in a dose‐dependent manner.

Alveoli are the terminal structure of the distal airway. Hematoxylin and eosin (H&E) staining (Figure 1c) showed that large numbers of epithelial cells and activated inflammatory cells (macrophages, neutrophils, and T lymphocytes) are released into the pulmonary environment, as previously reported (Strzelak et al., 2018). Alveolar macrophages are not mature, but the number of alveolar macrophages is increased significantly, which may lead to functional defects in alveolar macrophages (Arnson et al., 2010). Meanwhile, cigarette smoke caused increased alveolar wall thickness, plaque hemorrhage in lung tissue, and alveolar interstitial edema. However, emphysema due to smoking is characterized by the destruction of the alveolar wall, making it more susceptible to collapse and leading to further restriction of gas exchange, consistent with the literature (Rom et al., 2013). The corresponding Smith lung injury score (Figure 1d) showed reductions of 42%, 19%, and 62%, respectively, in the positive drug, low‐dose EBN, and high‐dose EBN groups as compared with the model group (*p* < .01).

### 3.2 EBN regulates systemic and pulmonary inflammatory factors caused by cigarette smoke

To further evaluate the effect of EBN on cigarette smoke‐induced lung inflammation, we first examined the serum inflammatory factors in cigarette smoke‐induced model mice. Compared with the control group, the model group had significantly increased serum levels of the pro‐inflammatory cytokines IL‐6 and TNF‐α (*p* < .01). The level of the anti‐inflammatory factor IL‐10 was significantly reduced in the positive drug, low‐dose EBN, and high‐dose EBN groups (*p* < .01). Compared with the model group, the serum levels of IL‐6 and TNF‐α in the positive drug, low‐dose EBN, and high‐dose EBN groups were reduced by 93%, 18%, and 53% and by 84%, 31%, and 70%, respectively. Meanwhile, in the positive drug, low‐dose EBN, and high‐dose EBN groups, the serum level of IL‐10 was increased by 100%, 160%, and 120%, respectively. This suggests that EBN alleviated systemic inflammation in the body in a dose‐dependent manner.

Furthermore, as smoking causes direct harm to the lungs, we investigated whether EBN might reduce inflammation at the site of injury. We discovered that EBN decreased the infiltration of inflammatory cells in lung tissue (Figure 1b). Therefore, we examined the pulmonary cytokines to elucidate the effect of EBN on airway inflammation caused by cigarette smoke. Similar to the results in serum, high‐dose EBN was found to reduce the level of the pro‐inflammatory cytokine TNF‐α in the lungs by 69% (*p* < .01). High‐dose EBN reduced the level of IL‐8 in the lungs by 45% (*p* < .05), while low‐dose EBN reduced the level of IL‐8 in the lungs by 23%. This implies that EBN alleviated the inflammatory response at the site of lung injury.

### 3.3 EBN regulates inflammatory factors by reducing the expression of pulmonary NLRP3 inflammasomes

To elucidate the changes in serum and lung cytokine levels, we investigated the upstream inflammasome involved in regulating the production of inflammatory factors. Immunohistochemical staining of the NLRP3 inflammasome in lung sections (Figure 3a) showed that EBN could inhibit the smoke‐induced increase in NLRP3 expression, with the high dose of EBN exerting a stronger inhibitory effect. The positive area score based on immunohistochemical staining (Figure 3b) showed that the cigarette smoke model increased NLRP3 inflammasome expressed in the lungs of mice by 105% (*p* < .01), while in the positive drug group, NLRP3 inflammasome expression was increased by only 45% (*p* < .01), and in the high‐dose EBN group, NLRP3 inflammasome expression was increased by 60% (*p* < .01). The NLRP3 expression level was not significantly different between the positive drug and high‐dose EBN groups. Consequently, our findings imply that EBN attenuated the inflammatory response by reducing the expression of the NLRP3 inflammasome.

### 3.4 EBN induces repair of the damaged alveolar epithelial layer by promoting the proliferation and viability of epithelial cells

**FIGURE 2:**
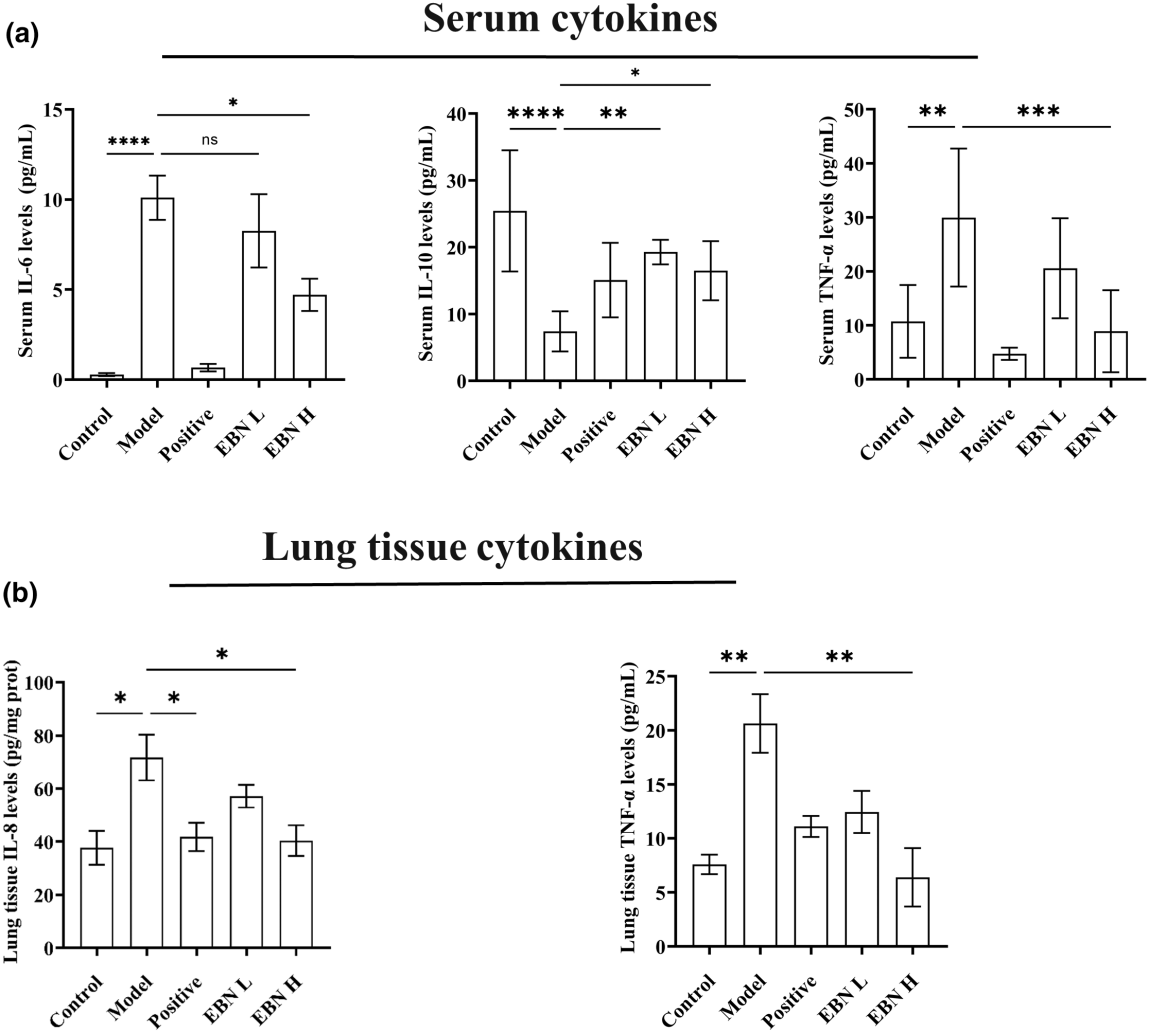
Ingestion of EBN can reduce the levels of inflammatory factors after cigarette smoke exposure. (a) The expression levels of IL‐6, IL‐10, and TNF‐α in serum. Three independent experiments were performed. Data are expressed as mean ± SD. Statistical analysis was conducted using nonparametric one‐way ANOVA. (b) The expression levels of IL‐8 and TNF‐α in lung tissue. Three independent experiments were performed. Data are expressed as mean ± SD. Statistical analysis was conducted using nonparametric one‐way ANOVA. Con, the control group; Model, the model group; Positive, the positive drug group (0.4 mL/10 g.bw); EBN L, the low‐dose EBN group (0.008 g EBN); EBN H, the high‐dose EBN group (0.019 g). *p ≤ .05, **p ≤ .01, ***p ≤ .001, ****p ≤ .0001.

**FIGURE 3:**
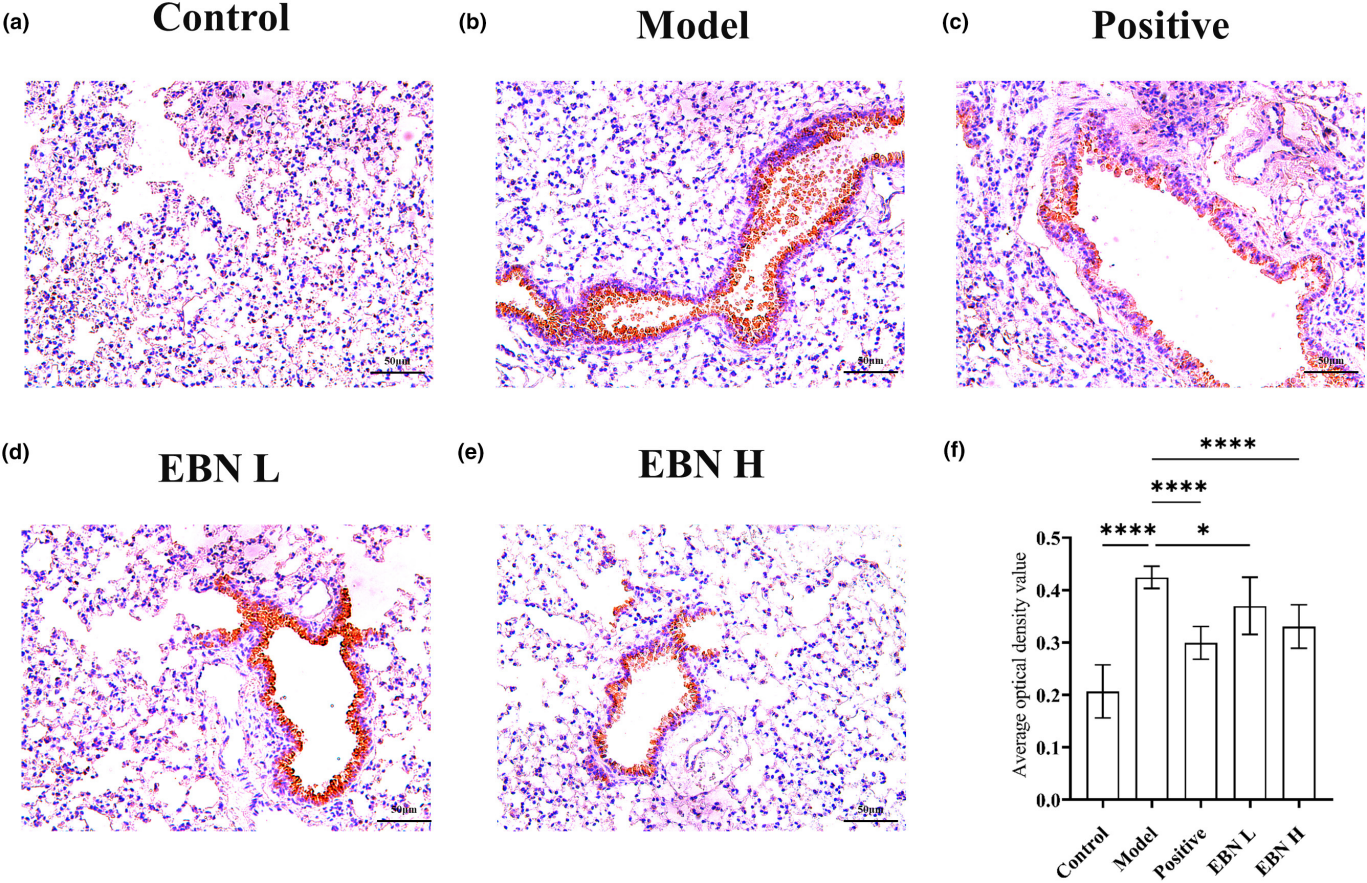
Ingestion of EBN can reduce NLRP3 inflammasome expression. (a–e) Immunohistochemical (IHC) staining of lung tissue. Magnification, 200×. Scale bar = 50 μm. (f) NLRP3 expression levels were semiquantitatively determined using the positive area score method; *n* = 10. Data are presented as mean ± SD. Statistical analysis was conducted using nonparametric one‐way ANOVA. Con, the control group; Model, the model group; Positive, the positive drug group (0.4 mL/10 g.bw); EBN L, the low‐dose EBN group (0.008 g EBN); EBN H, the high‐dose EBN group (0.019 g). *p ≤ .05, ****p ≤ .0001.

Tumor necrosis factor‐α (TNF‐α) intervention is a common way to model pneumonia because TNF‐α in lung tissue has a great effect on mortality in mice with lung inflammation (McGeough et al., 2017) (Figure 2b). We treated A549 cells with TNF‐α. The fully digested EBN has good nutritional characteristics (Wong, Chan, Wu, Lam, et al., 2018; Wong, Chan, Wu, Poon, et al., 2018), containing free sialic acid and small peptides, which can be absorbed into the blood (Chun et al., 1999; Tran et al., 2021) and reach the lungs. To model the complex changes to EBN upon ingestion, we processed EBN in vitro to simulate digestion in the stomach and small intestine and then treated A549 cells with simulated digested EBN. As with animal studies, we first treated cells with EBN for a period to form a preventive ‘system’ (Figure 3a).

**FIGURE 4:**
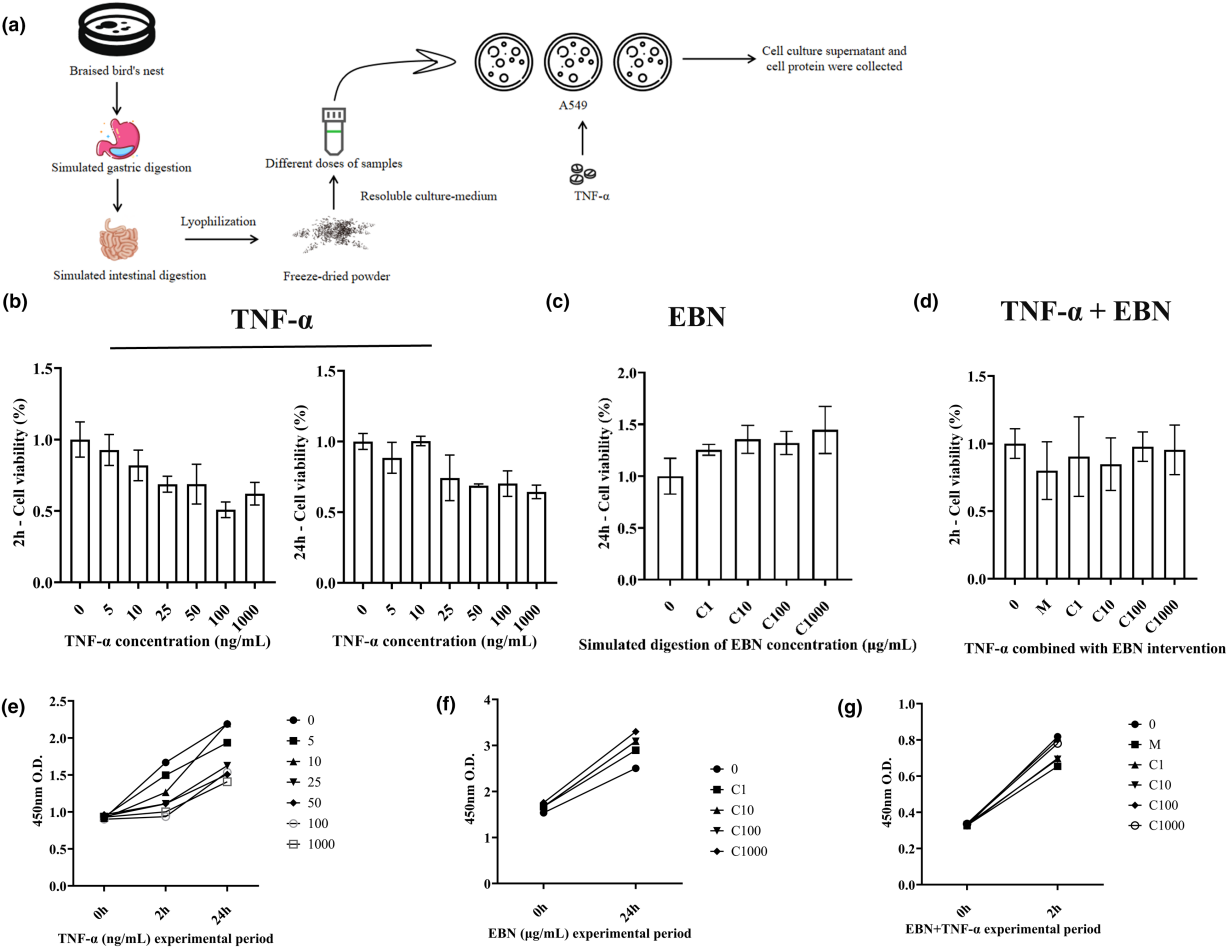
Effects of simulated digested EBN on cell proliferation. (a) Schematic of the experiment. A pneumonia model was established using A549 cells. (b–d) CCK8 assays were performed after treatment with (b) TNF‐α, (c) EBN, and (d) TNF‐α combined with EBN. Three independent experiments were performed. Data are expressed as mean ± SD. (e–g) Cell proliferation curves of e TNF‐α, f EBN, and g TNF‐α combined with EBN.

We first used the CCK8 cell proliferation assay to determine the suitable concentration of TNF‐α (Figure 4b). The activity of A549 cells was above 80% at a dose of 10 ng/mL, and a dose of 10 ng/mL for 2 h was selected for subsequent experiments. After that, because A549 cells must be treated with EBN for 24 h, we tested the toxicity of EBN on A549 cells (Figure 4c). The results showed that EBN did not cause cell toxicity but could significantly promote cell proliferation (*p* < .05). EBN at 1000 μg/mL increased cell proliferation 1.4‐fold.

After EBN pretreatment, stronger cellular activity was observed. To demonstrate that this prevention system could alleviate alveolar epithelial damage, TNF‐α was added after early EBN treatment, and the CCK8 assay was conducted. Upon combined treatment with EBN and TNF‐α (Figure 4d), EBN could relieve the cell damage caused by inflammatory conditions, and the cell viability in all treatment groups was over 80%. In conclusion, CCK8 assays showed that the simulated digested EBN not only did not cause toxicity to cells but even promoted the proliferation and viability of epithelial cells to alleviate the damage of the alveolar epithelial layer during treatment.

### 3.5 EBN regulates the expression of inflammatory factors in an in vitro model

**FIGURE 5:**
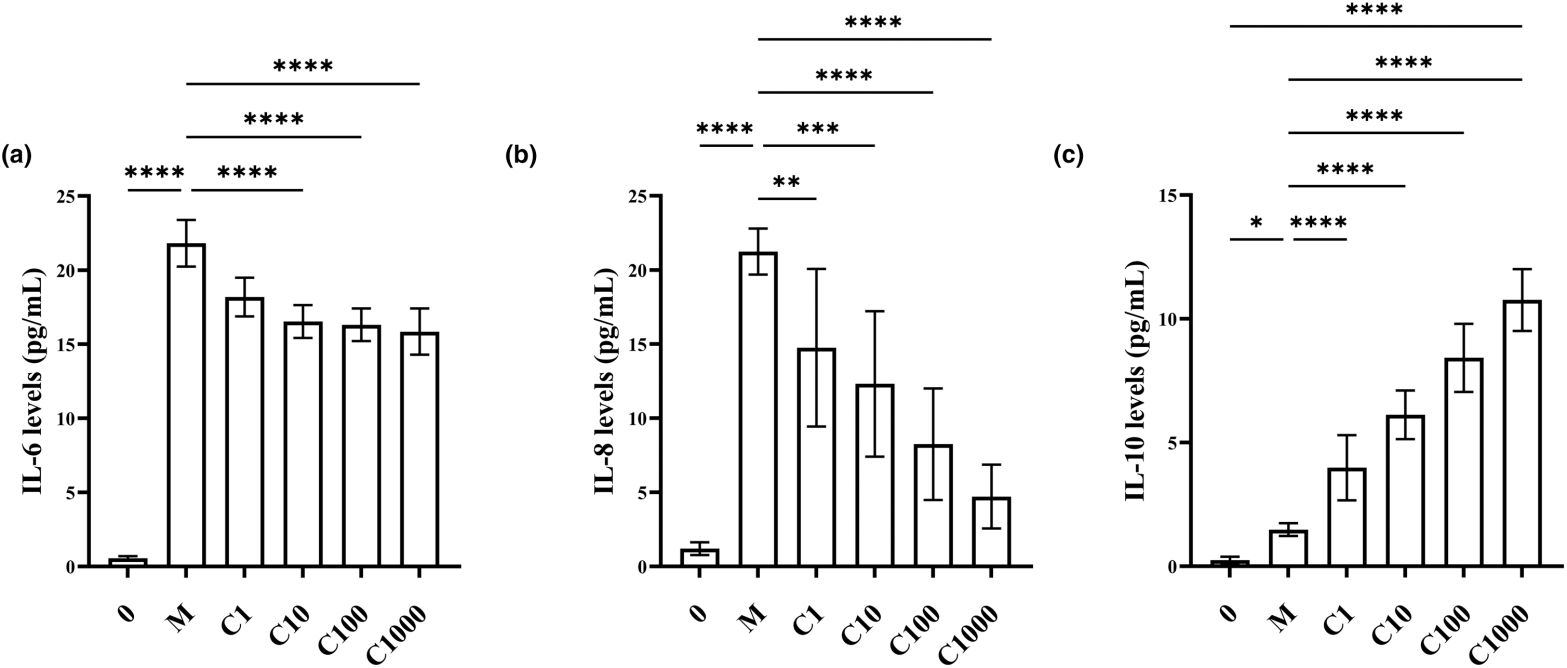
Simulated digested EBN regulates inflammatory factors in A549 cells. (a–c) The expression levels of inflammatory cytokines a IL‐6, b IL‐8, and c IL‐10. Three independent experiments were performed. Data are expressed as mean ± SD. Statistical analysis was conducted using nonparametric one‐way ANOVA. *p ≤ .05, **p ≤ .01, ***p ≤ .001, ****p ≤ .0001.

**FIGURE 6:**
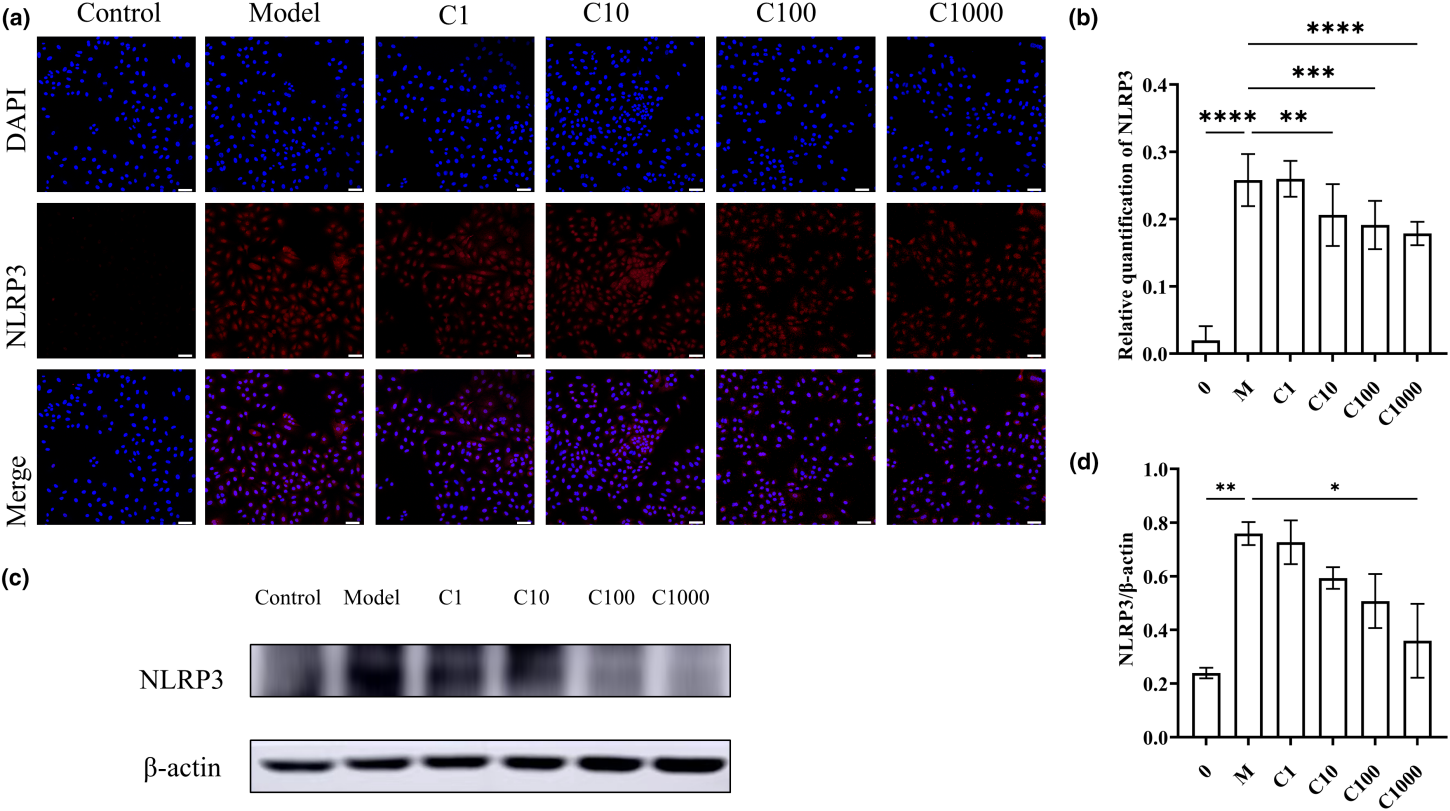
Simulated digested EBN alleviates NLRP3 inflammasome expression in A549 cells. (a) Cellular immunofluorescence staining of NLRP3. Scale bar = 50 μm. (b) Relative NLRP3 expression was quantified, according to the ratio of NLRP3 fluorescence intensity to DAPI fluorescence intensity. Data are presented as mean ± SD. Statistical analysis was conducted using nonparametric one‐way ANOVA. (c) The expression levels of NLRP3 and β‐actin were detected using Western blot analysis. (d) β‐Actin was used as the internal reference. Relative protein expression levels were determined using gray scale analysis. Three independent experiments were performed. Data are presented as mean ± SD. Statistical analysis was conducted using nonparametric one‐way ANOVA. *p ≤ .05, **p ≤ .01, ***p ≤ .001, ****p ≤ .0001.

In addition to regulating cytokines in animal experiments, cytokines in the supernatants of cultured cells were measured using ELISA. Because IL‐10 is not present in the supernatant of A549 cells (Holownia et al., 2016), protein lysates from A549 cells were used for the assay. Cytokines were almost not expressed in normal noninflammatory A549 cells (Figure 5a–c). Our results were consistent with those of Holownia et al. (Holownia et al., 2016). After TNF‐α treatment, the levels of IL‐6, IL‐8, and IL‐10 were increased 38‐fold (*p* < .01), 17‐fold (*p* < .01), and 5‐fold (*p* < .01), respectively. A concentration of 1000 μg/mL EBN had the most significant effect on the levels of inflammatory factors compared with the model group, reducing the IL‐6 level by 27% (*p* < .01), reducing the IL‐8 level by 78% (*p* < .01), and increasing the IL‐10 level 6.3‐fold (*p* < .01). In conclusion, we found that simulated digested EBN could decrease the levels of the pro‐inflammatory cytokines IL‐6 and IL‐8 while increasing the level of the anti‐inflammatory cytokine IL‐10 in a dose‐dependent manner (Figure 6).

### 3.6 EBN regulates inflammatory factors by reducing NLRP3 inflammasome expression in an in vitro model

To verify that the effects of EBN on the levels of inflammatory factors in vitro are mediated by the NLRP3 inflammasome, immunofluorescence staining and Western blot analysis were conducted. The NLRP3 inflammasome was almost not expressed in normal A549 cells without inflammation. After the cells were stimulated, NLRP3 expression was significantly increased (*p* < .01), and EBN decreased the expression of NLRP3 in a dose‐dependent manner. When the concentration of EBN was 1000 μg/mL, the expression of NLRP3 was inhibited by 31% compared with the model group (*p* < .01). Western blot analysis of protein lysates from lung tissue also showed that EBN reversed the increase in NLRP3 expression in a dose‐dependent manner. In conclusion, EBN inhibited NLRP3 expression in vitro.

### 3.7 EBN regulates pulmonary inflammation by regulating the NF‐κB pathway

**FIGURE 7:**
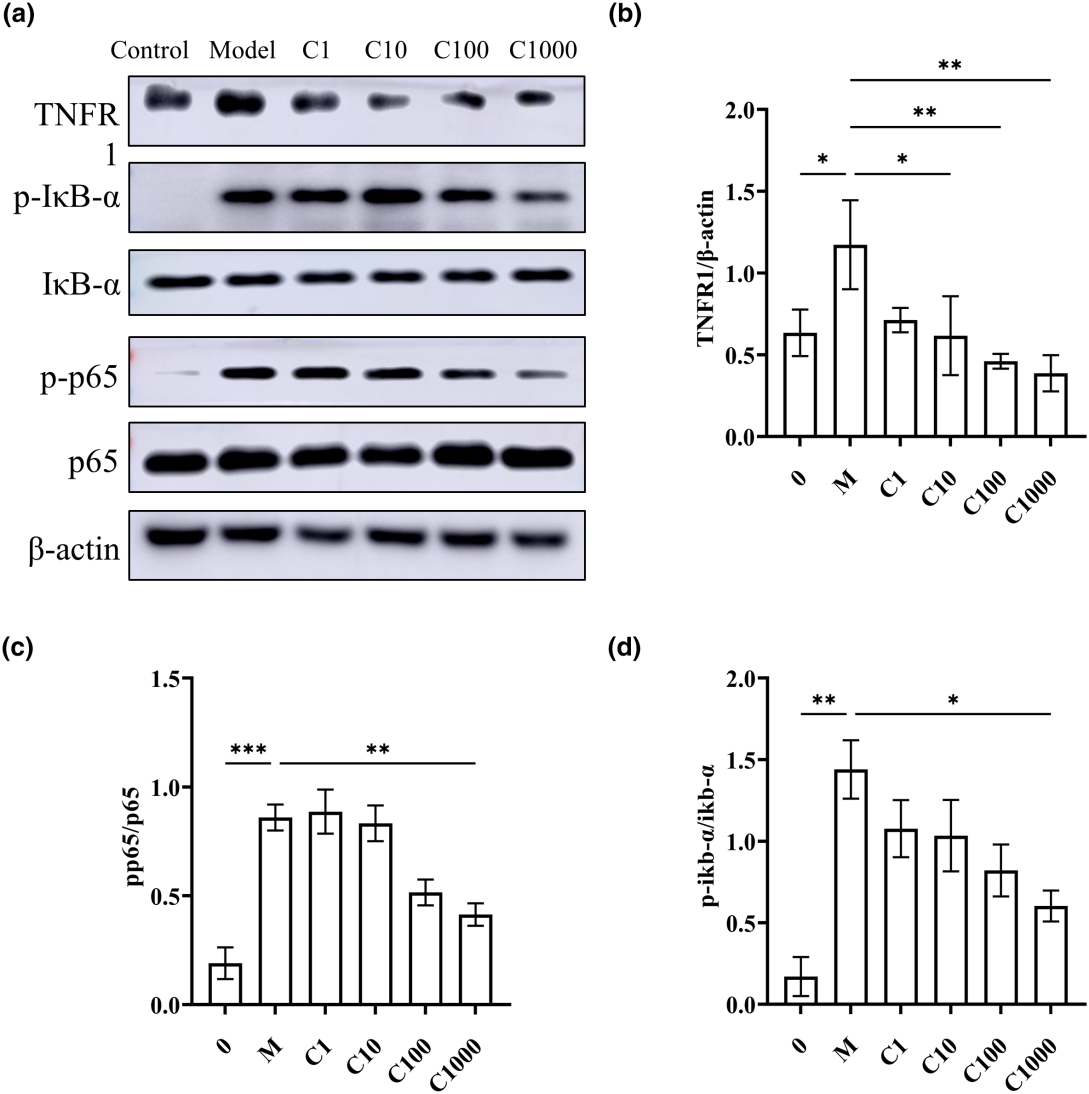
Exploring the mechanism by which EBN alleviates A549 cell inflammation. (a) The expression levels of TNFR1, p‐IκBα, IκBα, p‐NF‐κB p65, NF‐κB p65, and β‐actin were detected using Western blot analysis. (b) β‐Actin was used as the internal reference for TNFR1. (c) p65 was used as the control for p‐p65. (d) a p‐IκBα was used as the control for IκBα. Relative protein expression levels were determined using gray scale analysis. Three independent experiments were performed. Data are presented as mean ± SD. Statistical analysis was conducted using nonparametric one‐way ANOVA. *p ≤ .05, **p ≤ .01, ***p ≤ .001.

**FIGURE 8:**
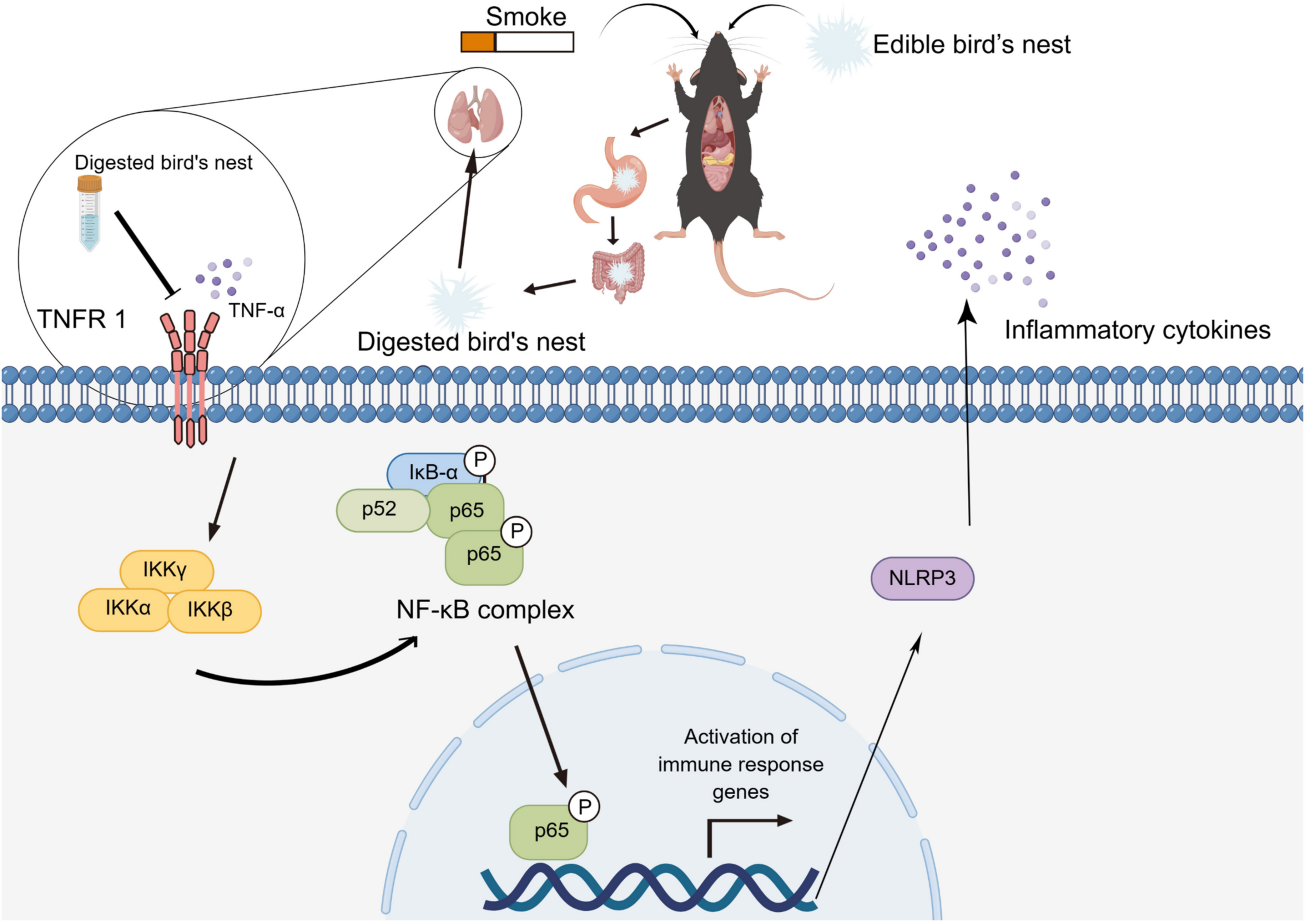
Schematic of the NF‐κB pathway.

Tumor necrosis factor receptor 1 (TNFR1) can trigger the classical inflammatory response upon binding with TNF‐α, inducing the phosphorylation of NF‐kappa B inhibitor alpha (IκBα) and activating the NF‐κB pathway. Because both the classical and nonclassical NF‐κB pathways require IκB kinase (IKK) for IκB activation (Scheidereit, 2006), we directly examined the levels of phosphorylated IκBα (Figure 7a). The Western blot results (Figure 7a) showed that compared with the control group, TNF‐α significantly increased the expression of TNFR1, which in turn significantly promoted the phosphorylation and degradation of IκBα. In addition, the phosphorylation of p65, a marker of the NF‐κB pathway, was promoted. However, after treatment with increasing concentrations of EBN, the expression of TNFR1 was reduced, and subsequently, the levels of activated phospho‐p‐IκBα (p‐IκBα) and phospho‐p65 (p‐p65) were gradually reduced. These findings suggest that EBN inhibited the phosphorylation of IκBα directly by inhibiting the binding of TNFR1 with TNF‐α in a dose‐dependent manner, blocking p65 phosphorylation, and thus downregulating the inflammatory response (Figure 8).

## 4 DISCUSSION

Currently, there is still no effective way to prevent the harm caused by tobacco use (Reitsma et al., 2021). Previous studies have shown that active smokers can be treated with nicotine replacement therapy, varenicline, and ibuprofen (Benipal et al., 2021), but there is still no suitable method to prevent the harm of secondhand smoke. It has recently been shown that dietary restriction has a protective effect on pulmonary inflammation (Mercader‐Barceló et al., 2020). EBN is used in traditional Chinese medicine to treat lung diseases (Haghani et al., 2016). In the present study, we investigated the effects of oral administration of EBN on the prevention or reduction of pulmonary inflammation in mice exposed to cigarette smoke. To the best of our knowledge, the present study is the first to explore the effect of oral EBN on pulmonary inflammation induced by cigarette smoke exposure and its mechanism of action.

We first demonstrated that EBN improves lung damage and inhibits the inflammatory responses induced by cigarette smoke. Strzelak et al. (2018) demonstrated that epithelial cells and activated inflammatory cells (macrophages, neutrophils, and T lymphocytes) are stimulated by cigarette smoke, released into the lung environment, and continuously recruited to the injury site, which is consistent with our results. Inflammation is a basic pathological process that occurs when biological tissues are stimulated by trauma, infection, and other factors, and it is a dynamic process of injury, anti‐injury mechanisms, and repair (Mack, 2018). In the model group, we found that mice with severe lung injury have more inflammatory cells at the site of severe injury. However, EBN intervention (especially high‐dose EBN) effectively reduced the degree of injury and the infiltration of inflammatory cells, which was consistent with the recent evidence showing that EBN inhibits inflammation and regulates the immune balance in mice with lung injury (Zeng et al., 2022). The recruitment of inflammatory cells by cigarette smoke may be caused by the activation of reactive oxygen species (ROS) and reactive nitrogen species (RNS) in inhaled tobacco smoke, and the interaction between cigarette smoke and endothelial cells will also lead to further generation of ROS in the airway (Barnes, 2022). Therefore, inhibiting inflammation is essential for the prevention of lung diseases in people exposed to cigarette smoke (Liu et al., 2023).

Furthermore, we showed that EBN inhibits the increase in the levels of TNF‐α and IL‐8, which serve as markers of smoking‐induced lung inflammation (Arnson et al., 2010; Rom et al., 2013), and increases the levels of the anti‐inflammatory factor IL‐10, whose levels are reduced by smoking. As a sensitive indicator reflecting the control of infection and inflammation in the body, IL‐6 expression increases significantly during inflammation or infection but rapidly decreases to normal levels after the control of inflammation (Hunter & Jones, 2015). We demonstrated that EBN inhibited the increase in serum IL‐6 levels and that serum TNF‐α levels were also reduced. This suggests that EBN alleviates lung inflammation and reduces the risk of autoimmune diseases, such as rheumatoid arthritis, systemic lupus erythematosus, and Graves' disease (Arnson et al., 2010). However, in a study by Roh et al. (2011), EBN increased the gene expression of IL‐6 and vascular endothelial growth factor (VEGF) to induce the proliferation of human adipose‐derived stem cells, in contrast to our findings, which may be related to the complex physiological functions of IL‐6, such as its proliferative activity (Hunter & Jones, 2015). After long‐term and repeated smoking, progressive inflammatory damage occurs in the body, and TNF‐α plays a leading role in the mortality of mice with lung inflammation (Freedman et al., 2002; Kim et al., 2009). TNF‐α is mainly produced by macrophages after cigarette stimulation (McGeough et al., 2017). Because our focus was on an in vitro model of lung parenchymal cells, we directly used TNF‐α to cause inflammation, according to the method of Tang et al. (2014). In the A549 cell pneumonia model, we confirmed that EBN decreased pro‐inflammatory factors while increasing anti‐inflammatory factors, which is consistent with the reports on the anti‐inflammatory effects of EBN (Lai et al., 2022; Wang et al., 2023; Yida et al., 2015; Zeng et al., 2022). In the present study, we show for the first time, to the best of our knowledge, that simulated digested EBN promotes epithelial cell proliferation and viability, which may help explain the reduced alveolar epithelial damage and lower levels of inflammation in the lungs and body.

In animal experiments, we found that the group with high levels of TNF‐α was also characterized by high NLRP3 inflammasome expression, which was consistent with the results of McGeough et al. (2017). TNF‐α is an important transcriptional regulator of the NLRP3 inflammasome in inflammatory diseases in mice (McGeough et al., 2017). Moreover, sustained activation of the NLRP3 inflammasome is associated with increased chemokine expression, recruitment of neutrophils and macrophages, and sustained production of interleukin 17A (IL‐17A) and TNF‐α (Wree et al., 2018). The NLRP3 inflammasome plays a key role in amplifying the inflammatory response and can stimulate the maturation of a large number of pro‐inflammatory cytokines (He et al., 2016), which corresponds to the results observed in our animal experiments: high NLRP3 inflammasome expression is associated with high levels of pro‐inflammatory cytokines. Recently, some studies showed that compared with the control mice, NLRP3^−/−^ mice did not show smoke inhalation‐related lung injury or IL‐1β secretion and showed lower inflammatory cell infiltration in the lungs (Yang et al., 2015). This is consistent with our observation of increased NLRP3 inflammasome expression after cigarette stimulation, which also suggests that inhibition of NLRP3 can alleviate the lung inflammatory response caused by cigarette smoke. According to our results, EBN effectively inhibits NLRP3 inflammasome expression. To the best of our knowledge, this is the first study to report the regulatory effect of EBN on the NLRP3 inflammasome.

Nuclear factor‐kappa B (NF‐κB) is known to act upstream of NLRP3 (Peng et al., 2020). By examining the NF‐κB pathway signaling proteins p‐IκBα and p‐p65, we showed that cigarette smoke stimulation activates the NF‐κB pathway in mouse lungs. Cigarette smoke compounds have been shown to activate the NF‐κB pathway in a variety of cells of the immune system, thereby inducing the activation of inflammatory cells (Gonçalves et al., 2011; Rom et al., 2013). Recently, Lai et al. (2022) reported that EBN reduces activation of the NF‐κB pathway signaling proteins p‐IκBα and p‐p65 in HaCaT keratinocytes in dermatitis models, which correspond to our results. To the best of our knowledge, the present study is the first study in which EBN has been shown to act on a signaling pathway in alveolar basal epithelial cells. EBN has also been shown to inhibit NF‐κB transcriptional activity in an in vitro inflammatory model of RAW264.7 cells stimulated by LPS (Li et al., 2023). However, according to the results reported by Roh et al. (2011), EBN can activate NF‐κB and activating protein‐1 (AP‐1) under certain circumstances, thereby increasing the gene expression of IL‐6 and VEGF to induce the proliferation of human adipose‐derived stem cells. Therefore, in this study, we demonstrated that EBN reduces the activation of the NLRP3 inflammasome by inhibiting the activation of the NF‐κB pathway during inflammatory injury.

However, Peng et al. (2020) recently reported that NLRP3 overexpression also promotes the activation of the NF‐κB pathway. According to our results, without the intervention of EBN, cigarette smoke can easily cause chronic and progressive inflammatory responses in the body. Therefore, inhibition of sustained activation of the NF‐κB pathway is very important. However, the mechanism responsible for the sustained activation of NF‐κB and NLRP3 inflammasome signaling is unknown (Henderson et al., 2020). TNFR1 is known to bind with TNF‐α and act as an upstream receptor to activate the NF‐κB pathway, thereby exacerbating inflammation (Zhang et al., 2020). In the present study, we found that TNFR1 is also expressed to a certain extent under normal physiological conditions, which is consistent with the results reported by Zhang et al. (2020). We further found that the expression of TNFR1 in alveolar basal epithelial cells was increased in the inflammatory state, while the expression of TNFR1 was inhibited after treatment with EBN. To the best of our knowledge, this is the first study showing that EBN inhibits TNFR1 expression in the inflammatory state, thereby reducing its binding with TNF‐α, which explains the inhibition of the activation of the NF‐κB pathway. Given the high cost and strong side effects of currently used TNF‐α inhibitors (Zhang et al., 2020), EBN shows good potential for developing next‐generation anti‐TNF‐α biologics.

In conclusion, in this study, we report for the first time, to the best of our knowledge, that EBN significantly improves cigarette smoke‐induced lung inflammatory responses via the TNFR1/NF‐κB/NLRP3 signaling pathway. Therefore, this study reveals a potential mechanism by which EBN alleviates pneumonia, providing a potential method for the prevention of injury caused by cigarette smoke and also new ideas for the application of EBN. However, EBN is a complex mixture of proteins, sialic acids, AMCase, polyunsaturated fatty acids (PUFAs), and other compounds, and simulated digested EBN contains small molecular anti‐inflammatory peptides (Wong, Chan, Wu, Lam, et al., 2018). The main active substances that exert anti‐inflammatory effects in EBN have not been elucidated. We plan to explore the active anti‐inflammatory substances of EBN in future studies.

## AUTHOR CONTRIBUTIONS

**Ran Bi:** Conceptualization (equal); data curation (equal); formal analysis (equal); investigation (equal); methodology (equal); project administration (equal); resources (equal); validation (equal); writing – original draft (equal); writing – review and editing (equal). **Dan Zhang:** Conceptualization (equal); data curation (equal); formal analysis (equal); validation (equal); writing – original draft (equal). **Rui Quan:** Data curation (equal); formal analysis (equal); investigation (equal); writing – original draft (equal). **Xiaoxian Lin:** Investigation (equal); methodology (equal); resources (equal); validation (equal); writing – original draft (equal). **Wen Zhang:** Data curation (equal); formal analysis (equal); investigation (equal); validation (equal); writing – original draft (equal). **Chuangang Li:** Data curation (equal); formal analysis (equal); investigation (equal); validation (equal); writing – original draft (equal). **Man Yuan:** Investigation (equal); methodology (equal); resources (equal); writing – original draft (equal). **Bing Fang:** Supervision (equal); validation (equal); writing – original draft (equal); writing – review and editing (equal). **Dongliang Wang:** Conceptualization (equal); methodology (equal); project administration (equal); supervision (equal); writing – original draft (equal); writing – review and editing (equal). **Yixuan Li:** Conceptualization (equal); data curation (equal); formal analysis (equal); funding acquisition (equal); methodology (equal); project administration (equal); validation (equal); writing – original draft (equal); writing – review and editing (equal).

## FUNDING INFORMATION

This work was supported by a grant from the 111 project of the Education Ministry of China (No. B18053).

## CONFLICT OF INTEREST STATEMENT

The authors declare that they do not have any conflict of interest.

## ETHICS STATEMENT

This study was approved by the ethical review regulations for animal experiments of Pony Testing International Group Co., Ltd. (Approval No. PONY‐2022‐FL‐13).

## INFORMED CONSENT

Written informed consent was obtained from all study participants.

## Data Availability

The data that support the findings of this study are available from the corresponding author upon reasonable request.
